# *BCR-ABL1* Doubling-Times and Halving-Times May Predict CML Response to Tyrosine Kinase Inhibitors

**DOI:** 10.3389/fonc.2019.00764

**Published:** 2019-08-13

**Authors:** Maria Stella Pennisi, Stefania Stella, Silvia Rita Vitale, Adriana Puma, Sandra Di Gregorio, Chiara Romano, Elena Tirrò, Michele Massimino, Agostino Antolino, Sergio Siragusa, Donato Mannina, Stefana Impera, Caterina Musolino, Giuseppe Mineo, Bruno Martino, Valentina Zammit, Francesco Di Raimondo, Livia Manzella, Fabio Stagno, Paolo Vigneri

**Affiliations:** ^1^Department of Clinical and Experimental Medicine, University of Catania, Catania, Italy; ^2^Center of Experimental Oncology and Hematology, Azienda Ospedaliera Universitaria (A.O.U.) Policlinico - Vittorio Emanuele, Catania, Italy; ^3^Department of Transfusional Medicine, Maria Paternò-Arezzo Hospital, Ragusa, Italy; ^4^Division of Hematology, Azienda Ospedaliera Universitaria (A.O.U.) Policlinico “P. Giaccone”, University of Palermo, Palermo, Italy; ^5^Division of Hematology, Papardo Hospital, Messina, Italy; ^6^Division of Oncology and Hematology, ARNAS Garibaldi-Nesima, Catania, Italy; ^7^Division of Hematology, Azienda Ospedaliera Universitaria (A.O.U.) Policlinico “G. Martino”, University of Messina, Messina, Italy; ^8^Division of Hematology, San Vincenzo Hospital, Taormina, Italy; ^9^Division of Hematology, Grande Ospedale Metropolitano, Reggio Calabria, Italy; ^10^Division of Hematology and Bone Marrow Transplant, Azienda Ospedaliera Universitaria (A.O.U.) Policlinico - Vittorio Emanuele, Catania, Italy; ^11^Department of Surgery, Medical and Surgical Specialties, University of Catania, Catania, Italy

**Keywords:** Chronic Myeloid Leukemia, doubling-time, halving-time, tyrosine kinase inhibitors, *BCR-ABL1/ABL1*^*IS*^

## Abstract

In Chronic Myeloid Leukemia (CML), successful treatment requires accurate molecular monitoring to evaluate disease response and provide timely interventions for patients failing to achieve the desired outcomes. We wanted to determine whether measuring *BCR-ABL1* mRNA doubling-times (DTs) could distinguish inconsequential rises in the oncogene's expression from resistance to tyrosine kinase inhibitors (TKIs). Thus, we retrospectively examined *BCR-ABL1* evolution in 305 chronic-phase CML patients receiving imatinib mesylate (IM) as a first line treatment. Patients were subdivided in two groups: those with a confirmed rise in *BCR-ABL1* transcripts without MR^3.0^ loss and those failing IM. We found that the DTs of the former patients were significantly longer than those of patients developing IM resistance (57.80 vs. 41.45 days, *p* = 0.0114). Interestingly, the DT values of individuals failing second-generation (2G) TKIs after developing IM resistance were considerably shorter than those observed at the time of IM failure (27.20 vs. 41.45 days; *p* = 0.0035). We next wanted to establish if decreases in *BCR-ABL1* transcripts would identify subjects likely to obtain deep molecular responses. We therefore analyzed the *BCR-ABL1* halving-times (HTs) of a different cohort comprising 174 individuals receiving IM in first line and observed that, regardless of the time point selected for our analyses (6, 12, or 18 months), HTs were significantly shorter in subjects achieving superior molecular responses (*p* = 0.002 at 6 months; *p* < 0.001 at 12 months; *p* = 0.0099 at 18 months). Moreover, 50 patients receiving 2G TKIs as first line therapy and obtaining an MR^3.0^ (after 6 months; *p* = 0.003) or an MR^4.0^ (after 12 months; *p* = 0.019) displayed significantly shorter HTs than individuals lacking these molecular responses. Our findings suggest that *BCR-ABL1* DTs and HTs are reliable tools to, respectively, identify subjects in MR^3.0^ that are failing their assigned TKI or to recognize patients likely to achieve deep molecular responses that should be considered for treatment discontinuation.

## Introduction

Unlike other solid or hematologic malignancies, Chronic Myeloid Leukemia (CML) is characterized by a single pathogenic alteration: the *BCR-ABL1* chimeric oncogene (1–6). *BCR-ABL1* is generated by a translocation involving the breakpoint cluster region (*BCR*) and the Abelson (*ABL1*) genes localized on chromosomes 22 and 9, respectively. The ensuing Philadelphia chromosome (Ph+) encodes for a constitutively active tyrosine kinase that promotes cell proliferation, modifies the actin cytoskeleton and modulates the interaction between leukemic cells and the bone marrow microenvironment (7–12).

While first- and second-generation (2G) tyrosine kinase inhibitors (TKIs) have generated unprecedented results for individuals diagnosed with chronic phase CML, solid evidence indicates that patients who do not achieve early molecular responses often display inferior outcomes, with increased risk of disease relapse, progression and death (13, 14). Hence, they require alternative forms of treatment (15). Branford et al. have previously shown that short *BCR-ABL1* doubling-times (DTs) (expressed in number of days) may be associated with CML progression to blast crisis (BC) (16). On the other hand, individuals who achieve a deep molecular response (MR^4.0^ or better) might be candidates for treatment discontinuation (17, 18).

Based on these data, we analyzed the DT of the *BCR-ABL1* transcripts in 305 chronic phase CML patients receiving Imatinib Mesllate (IM) as first line treatment to establish if different rises in *BCR-ABL1* transcripts could distinguish clinically negligible increases in oncogene levels from those indicative of TKI resistance. The 305 subjects were subdivided in two groups: those with a confirmed elevation in *BCR-ABL1* transcripts without major molecular response (MR^3.0^) loss, and those failing IM according to the latest European Leukemia Net (ELN) guidelines (19).

We also wanted to establish if a decrease in *BCR-ABL1* expression—defined as *BCR-ABL1* halving-time (HT)—could be employed to discern which CML patients were likely to achieve deep molecular responses and could be considered for TKI discontinuation. To this end, we analyzed the HTs of 174 patients receiving IM in first line, and analyzed their *BCR-ABL1* values at 6, 12, and 18 months of treatment.

Finally, as previous randomized phase III trials (20, 21) have suggested, employing 2G TKIs in place of IM as first line treatment for chronic phase CML induces faster and deeper clinical and molecular responses. Hence, we also analyzed the HT values of 50 patients receiving 2G TKIs as initial therapy for CML.

## Methods

### Patients and Treatment

Five hundred and twenty-nine early chronic phase CML patients were included in this study. Patients baseline characteristics are summarized in Supplementary Table 1. Subjects were followed in 10 Divisions of Hematology comprised in the SCREEN (Sicily and Calabria CML REgional ENterprise) Network from January 2005 to December 2018. Molecular monitoring was centralized in the Center of Experimental Oncology and Hematology of the A.O.U. Policlinico-Vittorio Emanuele. Four hundred seventy-nine patients received IM (400 mg/daily) whereas 50 were treated in first line with a 2G TKI. Specifically, 11 received dasatinib (DAS) 100 mg/daily while 39 received nilotinib (NIL) 300 mg twice/daily. Of the 479 individuals receiving IM, 305 were included for the calculation of *BCR-ABL1* DTs, while the remaining 174, the only individuals experiencing a continuous decrease of their oncogenic transcripts, were recruited to estimate *BCR-ABL1* HTs. Responses to therapy were evaluated according to the 2013 ELN criteria (19). The research ethics committee of each recruiting institution reviewed and approved the study protocol. The study was conducted according to the Helsinki Declaration.

### Molecular Response Definitions

*BCR-ABL1* transcripts were measured in peripheral blood samples, at diagnosis and every 3 months thereafter using Real-Time quantitative PCR (Q-PCR) as previously described (22–24). All collected samples were subjected to Q-PCR using the TaqMan platform and *ABL1* as a reference gene. Only individuals with the common e13a2 and/or e14a2 *BCR-ABL1* transcripts were included in our analysis. *BCR-ABL1* values were converted to the IS as previously described (24, 25). MR^3.0^ and MR^4.0^ were defined by *BCR-ABL1/ABL1*^IS^ values ≤0.1% and ≤0.01%, respectively, with no <10,000 *ABL1* copies (26).

For the DT and HT analysis, we considered patients with *BCR-ABL1/ABL1*^IS^ levels <10% since previous studies have shown that values ≥10% are quantitatively inaccurate (27). An increase of the *BCR-ABL1/ABL1*^IS^ transcript was defined as a >2-fold (if *BCR-ABL1/ABL1*^IS^ levels were ≥0.01%) or 5-fold (if *BCR-ABL1/ABL1*^IS^ levels were <0.01%) rise in two consecutive analyses (without therapeutic intervention).

For the HTs analysis, a reduction of *BCR-ABL1/ABL1*^IS^ transcripts was defined as a >2-fold (if *BCR-ABL1/ABL1*^IS^ levels were ≥0.01%) or 5-fold (if *BCR-ABL1/ABL1*^IS^ levels were <0.01%) decrease in two consecutive analyses.

### Doubling-Time and Halving-Time Calculation and Statistical Analyses

To calculate *BCR-ABL1* DTs we employed the following formula: DT = ln2/k, where (k) is the fold *BCR-ABL1* rise divided by the number of days over which the rise occurred [k = (ln(b) – ln(a))/d], with (a) the value before the rise, (b) the value at the rise, and (d) days (16). Similarly, the HT formula was calculated using the following formula: HT = -ln2/k, where (k) is calculated as reported above (28). Groups were compared using the *unpaired t*-test and the Kruskal-Wallis test employing the GraphPad software (version 5.0a).

## Results

### *BCR-ABL1* Doubling-Times Distinguish Patients With Negligible *BCR-ABL1* Increases From Those Failing IM

To establish if *BCR-ABL1* DTs could determine the clinical significance of a rise in the transcripts of the chimeric oncogene, we analyzed the evolution of *BCR-ABL1* mRNAs in 305 patients with chronic phase CML that had achieved a major molecular response (MR^3.0^) after receiving standard dose IM as first line treatment. The rates of treatment response of these individuals are summarized in Table 1.

**Table 1:** Rates of treatment responses.

	**DT IM first line (305 pz) (%)**	**HT IM first line (174 pz) (%)**	**HT 2G TKIs first line (50 pz) (%)**
CCyR	47.8	66.3	71.7
MMR	35.4	55.8	58.7
DMR	46.0	57.0	61.0
EFS	58.1	81.9	100
PFS	96.6	98.2	100
OS	91.3	92.4	95.6

*CCyR, complete cytogenetic response; MMR, major molecular response; DMR, deep molecular response; EFS, events free survival; PFS, progression free survival; OS, overall survival*.

**Figure 1:**
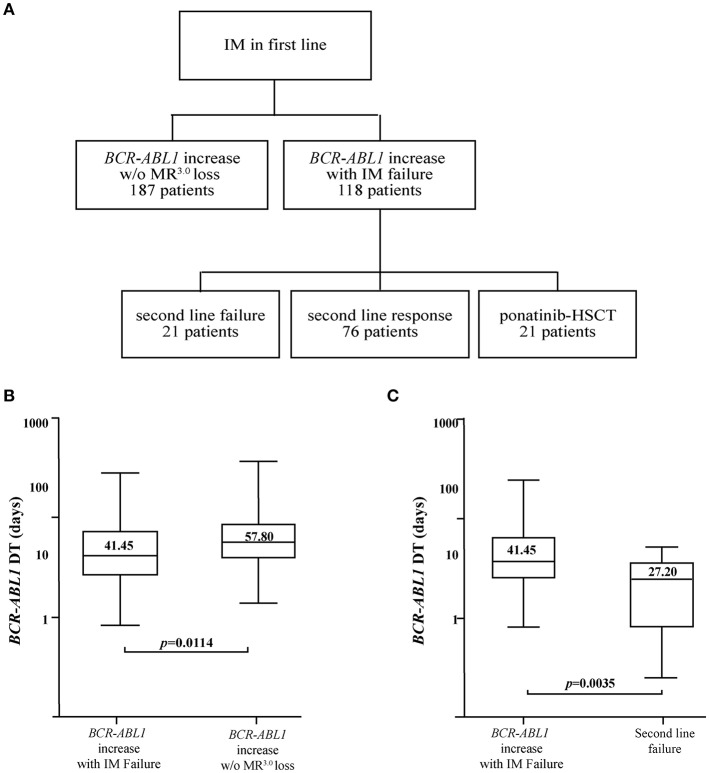
*BCR-ABL1* DTs in patients failing IM and 2G TKIs in second line. **(A)** Three hundred five patients with chronic phase CML presenting *BCR-ABL1/ABL1*^IS^ values ≤ 0.1% on IM therapy were included in this analysis. One hundred eighteen patients failed IM and switched to a different therapy, while 187 individuals displayed an increase in *BCR-ABL1* levels but retained their previously acquired MR^3.0^. Of the 118 individuals failing IM, 21 also failed the 2G TKI prescribed in second line while 76 responded to treatment. The remaining 21 patients either received ponatinib (*n* = 10) or were subjected to allogeneic human stem cell transplant (HSCT: *n* = 11). Boxplots delimited by the 25th (lower) and 75th (upper) percentile comparing *BCR-ABL1* DTs in the IM failure group and in subjects maintaining their MR^3.0^ **(B)** or in the IM failure group and in individuals failing 2G TKIs in second line **(C)**. Horizontal lines above and below each boxplot indicate the 5th and 95th percentile, respectively. Numbers inside each boxplot represent median values observed within each patient cohort. The reported *p*-values indicate statistical significance between the two patient groups included in each panel.

Of the 305 patients, 187 (61.3%) maintained their previously achieved MR^3.0^ despite an increase in *BCR-ABL1* levels, while 118 (38.7%) failed IM according to the 2013 ELN recommendations (Figure 1A). Statistical analyses determined that the *BCR-ABL1* DTs were significantly longer for individuals maintaining an MR^3.0^ as compared to those observed in subjects failing IM (57.80 vs. 41.45, *p* = 0.0114) (Figure 1B and Table 2).

**Table 2:** Comparison of *BCR-ABL1* DTs in patients with transcript increases but different clinical outcomes.

**Clinical context of *BCR-ABL1* rise**	**No of patients**	**DT (range)**	**Median *BCR-ABL1/ABL1*^**IS**^ before rise (range)**	**Median *BCR-ABL1/ABL1*^**IS**^ after rise (range)**	***p-*value**
*BCR-ABL1* increase with IM failure	118	41.45 (8.4–281.9)	0.076 (0.002–0.0943)	0.460 (0.110–48.93)	*p* = 0.0114
*BCR-ABL1* increase w/o MR^3.0^ loss	187	57.80 (14.3–356.2)	0.006 (0.00–0.031)	0.018 (0.002–0.100)	

Of the 118 individuals that failed IM, 50 received DAS, 47 received NIL, 11 (6 patients with T315I mutation and 5 subjects with compound mutations resistant to DAS and NIL) received ponatinib and 10 were subjected to an allogeneic stem cell transplant. Within the 97 individuals treated with a 2G TKI, 76 (78.3%) achieved an optimal response while 21 (21.7%) developed resistance to second line treatment (Figure 1A). We calculated the DTs for this last selected patient cohort and found a significant difference between the median DTs observed at the time of first or second treatment failure (41.45 vs. 27.20 days; *p* = 0.0035) (Figure 1C and Table 3).

**Table 3:** Comparison of *BCR-ABL1* DTs in patients failing first and second line treatment with IM and 2G TKIs.

**Clinical context of *BCR-ABL1* rise**	**No of patients**	**DT (range)**	**Median *BCR-ABL1/ABL1*^**IS**^ before rise (range)**	**Median *BCR-ABL1/ABL1*^**IS**^ after rise (range)**	***p-*value**
*BCR-ABL1* increase with IM failure	118	41.45 (8.4–281.9)	0.076 (0.002–0.0943)	0.460 (0.110–48.93)	*p* = 0.0035
Second line failure	21	27.20 (2.5–56.3)	0.149 (0.0002–5.85)	4.850 (0.48–20.12)	

These data suggest that DT analysis may discriminate clinically negligible increases in *BCR-ABL1* levels from those associated with IM failure. Our data also confirm that CML patients failing two lines of treatment have an aggressive and rapidly proliferating disease that requires urgent medical attention.

### *BCR-ABL1* Halving-Times Identify Patients Likely to Achieve Deep Molecular Responses With IM

Consolidated evidence suggests that CML patients achieving and maintaining deep molecular responses (≥MR^4.0^) may be considered for treatment discontinuation (29). To evaluate if *BCR-ABL1* HTs could be employed to identify individuals likely to obtain molecular responses ≥MR^4.0^, we analyzed the reduction in *BCR-ABL1* transcripts of 174 subjects receiving standard dose IM and selected three different time-points: 6, 12, and 18 months of treatment. The rates of treatment response for these patients are summarized in Table 1.

**Figure 2:**
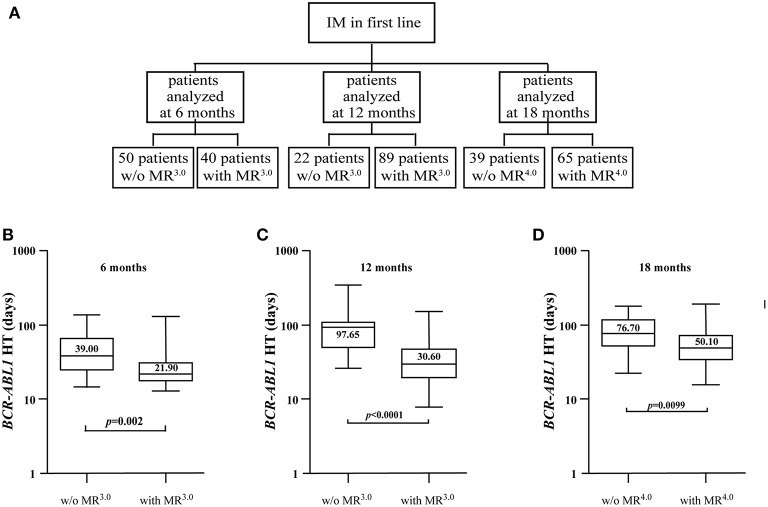
Differences in *BCR-ABL1* HTs between patients able or unable to achieve MR^3.0^ or MR^4.0^ at different time points. **(A)** Flow chart depicting *BCR-ABL1* HTs calculated, at different time points, for a total of 174 CML patients receiving standard dose IM. Boxplots delimited by the 25th (lower) and 75th (upper) percentile show HTs calculated after 6 **(B)**, 12 **(C)**, and 18 **(D)** months of IM. Horizontal lines above and below each boxplot indicate the 5th and 95th percentile, respectively. Numbers inside each boxplot represent median values observed within each patient cohort. The reported *p*-values indicate statistical significance between the two patient groups included in each panel.

As most patients were evaluated at multiple time points, the overall number of samples analyzed to calculate *BCR-ABL1* HTs was 305. Specifically, samples from 90 individuals were available after 6 months of IM (50 without MR^3.0^, 40 in MR^3.0^), 111 specimens were accessible after 12 months (22 without MR^3.0^, 89 in MR^3.0^) and 104 samples could be tested after 18 months (39 without MR^4.0^ and 65 in MR^4.0^) (Figure 2A). At the 6-month time point, the median HT for the 50 patients not in MR^3.0^ was 39 days (range 14.9–142.6) compared to 21.90 days (range 13.0–131.6) for the 40 individuals with an MR^3.0^. This difference was statistically significant (*p* = 0.002) (Figure 2B and Table 4). After 12 months of IM, we compared the *BCR-ABL1* HTs of the 22 patients without MR^3.0^ to those of the 89 individuals with MR^3.0^. Median HTs were significantly longer for patients without MR^3.0^ 97.65 days (range 26.4–354.3) as compared to individuals in MR^3^ 30.60 days (range 7.8–156.1) (*p* < 0.0001) (Figure 2C and Table 4). Similarly, after 18 months of IM, HTs in the 39 subjects in MR^3.0^ were 76.70 days (range 15.1–179.9), a significantly higher value than that recorded in the 65 patients in MR^4.0^ (50.10 days; range 15.5–192.2) (*p* = 0.0099) (Figure 2D and Table 4).

**Table 4:** Comparison of *BCR-ABL1* HTs at different time points in patients treated with IM and stratified according to the achieved molecular responses.

**Clinical context at time of *BCR-ABL1* decrease**	**No of samples analyzed**	**HT (range)**	**Median *BCR-ABL1/ABL1*^**IS**^ before the decrease (range)**	**Median *BCR-ABL1/ABL1*^**IS**^ after the decrease (range)**	***p-*value**
IM w/o MR^3.0^ by 6 months	50	39.00 (14.9–142.6)	1.760 (0.210–9.540)	0.267 (0.115–0.989)	*p* = 0.002
IM with MR^3.0^ by 6 months	40	21.90 (13.0–131.6)	0.524 (0.109–5.364)	0.033 (0.001–0.098)	
IM w/o MR^3.0^ by 12 months	22	97.65 (26.4–354.3)	1.320 (0.93–9.60)	0.621 (0.188–1.030)	*p* <0.001
IM with MR^3.0^ by 12 months	89	30.60 (7.8–156.1)	0.263 (0.112–4.244)	0.040 (0.001–0.098)	
IM w/o MR^4.0^ by 18 months	39	76.70 (15.1–179.9)	0.100 (0.021–1.002)	0.038 (0.011–0.099)	*p* = 0.0099
IM with MR^4.0^ by 18 months	65	50.10 (15.5–192.2)	0.011 (0.000–0.678)	0.003 (0.000–0.033)	

Hence, calculating *BCR-ABL1* HTs may prove of clinical value to rapidly recognize CML patients that are likely to achieve deep molecular responses on IM.

### *BCR-ABL1* Halving-Times Identify Patients Likely to Achieve Deep Molecular Responses With 2G TKIs

**Figure 3:**
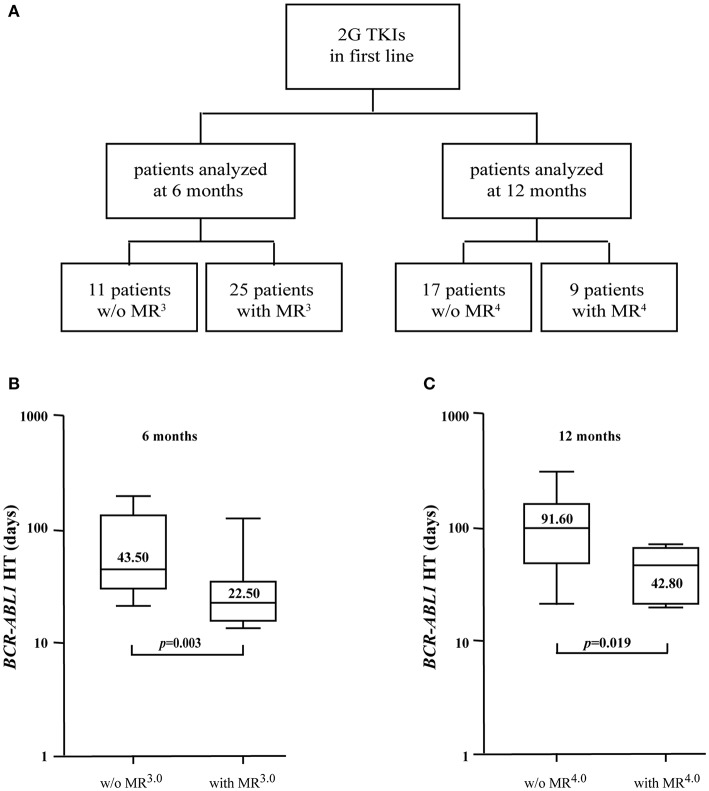
*BCR-ABL1* HTs in patients receiving 2G TKIs as first-line therapy. **(A)** *BCR-ABL1* HTs were calculated in a total of 62 samples evaluated after 6 (*n* = 36) or 12 (*n* = 26) months of treatment with 2G TKIs. Boxplots delimited by the 25th (lower) and 75th (upper) percentile indicate HTs comparing individuals with or without an MR^3.0^ after 6 months **(B)** or subjects with or without an MR^4.0^ after 12 months **(C)** of therapy. Horizontal lines above and below each boxplot indicate the 5th and 95th percentile, respectively. Numbers inside each boxplot represent median values observed within each patient cohort. The reported *p*-values indicate statistical significance between the two patient groups included in each panel.

To investigate the differences in the velocity of *BCR-ABL1* reduction in patients receiving 2G TKIs, we measured the HTs of 50 individuals treated in first line with DAS or NIL (Figure 3A). The rates of treatment response for these subjects are summarized in Table 1. In detail, patients without MR^3.0^ after 6 months of treatment had a *BCR-ABL1* HT of 43.50 days (range 21.4–195.1) vs. 22.50 days (range 13.2–116.6) for those in confirmed MR^3.0^ after 6 months (*p* = 0.003) (Figure 3B and Table 5). We next repeated this assessment on 26 patients that had received 2G TKIs for 12 months: 17 were without MR^4.0^ while 9 had attained an MR^4.0^. Median *BCR-ABL1* HTs were 91.60 days for the former patients (range 20.3–290.9) vs. 42.80 days for the latter group (range 19.8–66.5) (*p* = 0.019) (Figure 3C and Table 5).

**Table 5:** Comparison of *BCR-ABL1* HTs at different time points in patients treated with 2G TKIs and stratified according to the achieved molecular responses.

**Clinical context at time of *BCR-ABL1* decrease**	**No of samples analyzed**	**HT (range)**	**Median *BCR-ABL1/ABL1*^**IS**^ before the decrease (range)**	**Median *BCR-ABL1/ABL1*^**IS**^ after the decrease (range)**	***p-*value**
2G TKIs w/o MR^3.0^ by 6 months	11	43.50 (21.4–195.1)	1.515 (0.336–6.664)	0.250 (0.120–1.483)	*p* = 0.003
2G TKIs with MR^3.0^ by 6 months	25	22.50 (13.2–116.6)	0.451 (0.106–4.85)	0.046 (0.002–0.098)	
2G TKIs w/o MR^4.0^ by 12 months	17	91.60 (20.3–290.9)	0.086 (0.018–4.850)	0.031 (0.013–0.095)	*p* = 0.0019
2G TKIs w/o MR^4.0^ by 12 months	9	42.80 (19.8–66.5)	0.045 (0.011–3.083)	0.005 (0.003–0.010)	

These results confirmed the clinical usefulness of assessing *BCR-ABL1* molecular kinetics in CML patients receiving 2G TKIs.

## Discussion

Disease risk (measured with the Sokal, EURO or EUTOS Long-Term Survival score), *BCR-ABL1/GUS*^IS^ transcripts at diagnosis, individual comorbidities and expected patient compliance are good parameters to select between IM and 2G TKIs for the initial treatment of chronic phase CML (19). Moreover, reductions in *BCR-ABL1/ABL1*^IS^ transcripts are pivotal determinants of therapeutic efficacy regardless of the prescribed TKI, as indicated in the latest ELN guidelines (19). However, it is currently difficult to identify the correct therapeutic approach for the heterogeneous group of patients with a TKI response classified as “warning.”

As previously reported by Branford et al. (16), an increase in *BCR-ABL1* transcripts may be predictive of a potential loss of IM response. Specifically, in the Australian analysis, short (9 days) *BCR-ABL1* DTs were indicative of disease evolution to BC or of IM discontinuation/interruption, while longer (48 days) DTs were associated with TKI failure due to point mutations in the *BCR-ABL1* kinase domain without progression to BC. To support these results in a different patient cohort, we calculated the *BCR-ABL1* DTs of 305 CML patients receiving standard dose IM in first line. As our patient series was devoid of subjects progressing to BC, we could not confirm the findings of Branford et al. on the extremely brief DT detected in patients with disease evolution. However, in agreement with their results, we found that patients failing IM displayed DTs <42 days, a value extremely similar to that reported by Branford et al. These findings suggest that individuals with DTs of 42–48 days should be quickly considered for a change of therapy (Figure 1). In addition, to establish if IM failure correlated with alterations in the sequence of the *BCR-ABL1* kinase domain, we analyzed this patient cohort by clonal sequencing. We found resistant mutations (F317L, Y253H, M351T) in 3 patients and alternatively spliced variants (i.e., 35 bp insertion), in other 5 individuals (data not shown). Our results are in line with those previously reported by Yuda et al. showing that a 35 bp insertion might be correlated with loss of IM response (30).

In our cohort, patients failing IM were treated with 2G TKIs (94 in total) or received ponatinib (11 in total). These treatment decisions were guided by mutational analyses performed at the time of drug failure, as the 11 subjects given ponatinib displayed the T315I substitution (6 patients) or compound mutations (Y253H/F317L in 2 subjects, V299L/M351T in 3 patients) responsive to the drug, as previously reported by Zabriskie et al. (31).

Individuals failing both IM and a 2G TKI displayed a significantly lower *BCR-ABL1* DT at the time of second line failure (27.20 vs. 41.45 days; *p* = 0.0035) indicative of a biological evolution toward a more aggressive disease (Figure 1).

Several studies (17, 29, 32–34) have shown that patients who maintain undetectable minimal residual disease may be candidates for therapy discontinuation. However, the early identification of such patients remains a controversial issue. Hence, as suggested by Branford et al. (28), we analyzed the reduction in oncogenic transcripts after 6, 12, and 18 months of IM to calculate a *BCR-ABL1* HT and establish if this parameter is useful to recognize patients that should be considered for treatment cessation. Our data confirm that TKI-sensitive disease follows a biphasic decline (a rapid reduction during the first 6 months followed by a more gradual decline thereafter) as previously reported [(35); Figures 2, 3]. This pattern is likely attributable to an initial decrease in the number of differentiated Ph+ neutrophils followed by a reduction in the rate of leukemic stem cell turnover (28, 36, 37). Thus, the early (at 6 months) achievement of a major molecular response followed by a further *BCR-ABL1* decrease to a deep molecular response (≥MR^4.0^) may be considered a selective parameter favoring the rapid identification of individuals potentially eligible for TKI discontinuation.

Both the DASISION and the ENESTnd studies (20, 21) have suggested that the use of 2G TKIs as first line treatment for chronic phase CML induces faster and deeper responses than IM. To compare the *BCR-ABL1* decline in patients treated with IM or 2G TKIs, we measured the oncogene's HTs in individuals receiving DAS or NIL as first line treatment. As expected, we found no differences in the velocity of *BCR-ABL1* reduction between patients achieving the same molecular responses (MR^3.0^ at 6 months), regardless of the TKI they were receiving (21.90 vs. 22.50 days). However, we detected a sizeable difference in the number of subjects attaining this response: 44% in the IM group vs. 69% in the 2G TKI group. This finding confirms that individuals displaying excellent responses to IM will obtain a comparable benefit to that achievable with 2G TKIs but that the overall number of these subjects is clearly inferior to that attainable with DAS or NIL. These differences are amplified at later time points, with only 13% of IM-treated individuals achieving an MR^4.0^ at 12 months (data not shown) compared to 35% for those receiving 2G TKIs. Furthermore, from the 12-month time point onwards, *BCR-ABL1* HTs were constantly shorter in patients treated with 2G TKIs explaining the higher number of deep molecular responses observed with these drugs. However, our data suggest that, regardless of the type of inhibitor, after 12 months of TKI therapy a HT value of 40 days is associated with a higher probability of achieving a deep molecular response.

In summary, *BCR-ABL1* DTs and HTs are easily measurable molecular parameters that rely on the timely computation of the variations in *BCR-ABL1* transcripts that are routinely measured in real life CML monitoring. Both molecular indexes can be of great value in complex clinical situations i.e., in interpreting a rise in *BCR-ABL1* levels in patients that have achieved an MR^3.0^ and in discriminating patients that exhibit modest declines in their oncogenic transcripts from those who will likely achieve a deep molecular response and might therefore be eligible for TKI discontinuation.

## Data Availability

All datasets generated for this study are included in the manuscript and/or the Supplementary Files.

## Ethics Statement

The research ethics committee of each recruiting Institution reviewed and approved the study protocol (Data Sheet 1). The study was conducted according to the Helsinki Declaration.

## Author Contributions

MP, SSt, and SV contributed to design the study methodology, to carry out the laboratory work, to collect, and to interpret the data. MP wrote the draft manuscript and performed statistical analysis. AP and SD contributed to carry out the laboratory work and interpret the results. CR, ET, and MM contributed to interpret the results. AA, SSi, DM, SI, CM, GM, BM, and VZ recruited and followed the patients. LM contributed to interpret the results and critically reviewed the paper. FS, BM, and VZ recruited and followed the patients, interpreted the results, and critically reviewed the paper. FD interpreted the results and critically reviewed the paper. PV designed the study, supervised the project, contributed to the interpretation of the data, revised the manuscript draft, and critically reviewed the paper. All authors read and approved the manuscript in its present final form.

### Conflict of Interest Statement

FS honoraria from BMS, Incyte, Novartis, Pfizer. FD research funding from BMS, honoraria from Novartis, Incyte, and Pfizer. PV research funding from Astra-Zeneca and Novartis, honoraria from Astra-Zeneca, Celgene, Incyte, Novartis, Pfizer, Tesaro, and Teva. The remaining authors declare that the research was conducted in the absence of any commercial or financial relationships that could be construed as a potential conflict of interest.
