# Supplementary material for: BCR-ABL1 Doubling-Times and Halving-Times May Predict CML Response to Tyrosine Kinase Inhibitors
Source: Front Oncol. 2019 Aug 13;9:764. doi: 10.3389/fonc.2019.00764 (PMC6700306; doi:10.3389/fonc.2019.00764)
Supplement: Supplementary file 1 [file Table_1.pdf]

Supplementary Table 1. Patient Characteristics (N = 529)

Characteristics		
**Age**	**years**	
Median	55.4	
Range	17-90	
**Sex Distribution**	**n°**	**%**
Male	272	51.4
Female	257	48.6
**Hb**	**g/dl**	
Median	12.1	
Range	6.8-16	
**WBC**	**$*10^9/L$**	
Median	99.8	
Range	24.6-500	
**PLT**	**$*10^9/L$**	
Median	426.6	
Range	46-664	
**Splenomegaly**	**cm**	
Median	3.1	
Range	0-30	
**Additional Chromosomal Abnormalities (ACA)**	41 (n°)	7.7 (%)

**Sokal risk**	**n°**	**%**
Low/Int	444	83.9
High	85	16.1
**ELTS risk group**	**n°**	**%**
Low/Int	500	92.6
High	29	7.4
**Transcript Type**	**n°**	**%**
e13a2	229	43.2
e14a2	252	47.6
e13a2 and e14a2	48	9.2
